# First principles quantum calculations for graphyne for electronic devices

**DOI:** 10.1039/d1na00336d

**Published:** 2021-09-03

**Authors:** Xianwei Sha, Clifford M. Krowne

**Affiliations:** a General Dynamics Information Technology Corporation Falls Church VA 22042 USA; b Information Technology Division, Center for Computational Science, Naval Research Laboratory Washington DC USA; c Electromagnetics Technology Branch, Electronics Science & Technology Division, Naval Research Laboratory Washington DC 20375 USA cliffordkrowne@outlook.com

## Abstract

Moving beyond traditional 2D materials is now desirable in order to have switching capabilities (*e.g.*, transistors). Here we propose using γ graphyne-*n* because, as shown in this paper, obtaining regions of the electronic band structure which act as valence and conduction bands, with an apparent bandgap, are found. Electron spatial density and electronic band structures with *ε*(*k*) *vs. k* are calculated for graphyne-1 and graphyne-2 having respectively, one and two triple C–C carbon–carbon bonds between adjoining benzene rings; such side by side comparisons never before done. The *ab initio* quantum calculations were performed using both the local density approximation (LDA) and the generalized gradient approximation (GGA) for density functional theory (DFT).

## Abstract 2

Electronic band structure diagrams are found using DFT *ab initio* calculations with LDA and GGA for the graphyne allotropes graphyne-*n* with *n* = 1, 2, showing nominal bandgap values of 0.5 eV, acceptable for low and medium power electronics.

## Graphical abstract

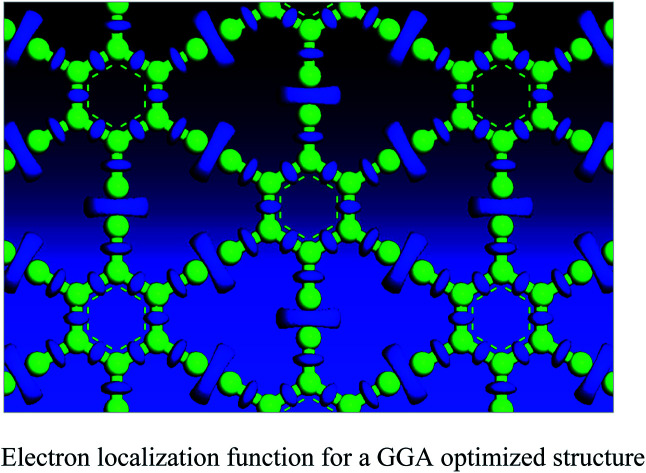

## 1. Introduction

For high frequency electronic devices, there are several passive and active structures that are often utilized.^1–5^ These include isolators, circulators, and phase shifters which are common control components. Also required are RF transmission structures, which include stripline, microstrip, slot line, and coplanar line. All of these structures may be miniaturized to some extent. Movement to much higher frequencies beyond the microwave, millimeter wave bands, into those frequency regimes heading toward 100 GHz, and well beyond that requires something be done to improve on the active transistor devices currently employed which utilize GaAs and GaN, their sister compounds, and other similar materials. All of these transistor type of RF materials are expensive to process, and possess often dangerous elements unfriendly to the environment, so there is a push to find other high speed materials, especially 2D materials for RF electronics.

Our focus will be on atomically planar like materials, because nanowire and nanotube alignment is known to be a difficult challenge with substantial fabrication costs.^6–8^ Lower dimensional materials (0D, 1D, and 2D) must be employed in future transistors because only atomic scale structures can improve the performance. The quantum approach accounting for many body interactions is adopted here, as opposed to more classical and semi-classical transport methods,^9–12^ since detailed band structure information is sought. For sought after transistor action, bandgaps *E*_g_ at or above a few tenths of an eV are required. Thus, *E*_g_ ≥ 0.25 eV, with the minimum for low power (*E*_g_ = 0.25 eV), materials like Si and GaAs for medium power (*E*_g_ = 1.12 and 1.42 eV), and SiC and GaN for high power (3.26 and 3.42 eV).^13,14^

Recently, we did address the monoatomic 2D material borophene, and determined its properties using a hybrid functional approach, investigating three types of hybrids.^15^ The interest in borophene arises because it is elemental, there are known methods for processing it, can be fabricated in a planar form, and shows some promise of finite electronic bandgaps. Here attention is turned toward a second monoatomic 2D material, graphyne, which also can possess flat sheets, and shows great promise of being processed with noticable electronic bandgaps.

Carbon in a higher local energy minima state compared to graphene, creates a new type of carbon based 2D material, graphyne. It shows promise as a high mobility transport material for electronics too, similar to graphene, but unlike pure graphene which has Dirac states at symmetry points in *k*-space and no bandgap, graphyne has finite bandgaps. Although the structures of graphyne are considerably more complicated than its sister material graphene, the intrinsic simplicity of dealing with a monoatomic material is tremendously attractive still. The idea of using monoatomic semiconductors to make individual transistors and eventually integrated circuits is not a new one. This began almost 70 years ago with their use in TVs, radios, electronic calculators and communication systems. Of course, back then, the monoatomic materials were germanium and silicon.

It is known that graphyne has allotropes which are planar, and the predicted electronic properties hold interest for high speed transport of carriers.^16–20^ Growth and preparation of the material for further exploitation in electronic devices, for example, require various preparation methods.

Graphyne allotropes display bandgaps appropriate for semiconductor electronics. The various allotropes of graphyne are hexagonal honeycomb lattices, but with their benzene rings connected by acetylene triple C–C carbon–carbon bonds. This makes for extra complicated requirements for not only performing density functional theory calculations (DFT), but also in their physical preparation in a laboratory environment. The band structure will be shown to be not gapless as in graphene with its characteristic Dirac type band structure with its relativistic analogy to low mass particles, but parabolic type with its more massive characteristic of semiconductor action (and a bandgap).

There are several advantages to developing a new semiconductor material for electronics based on carbon related to graphene, because of the extensive prior work done on graphene growth and preparation and its electronics. The unique characteristic for graphyne is that it consists of single and triple acetylene bonds, allowing creation of a material with attractive thermal and electrical transport properties.^16–18^

**Fig. 1:**
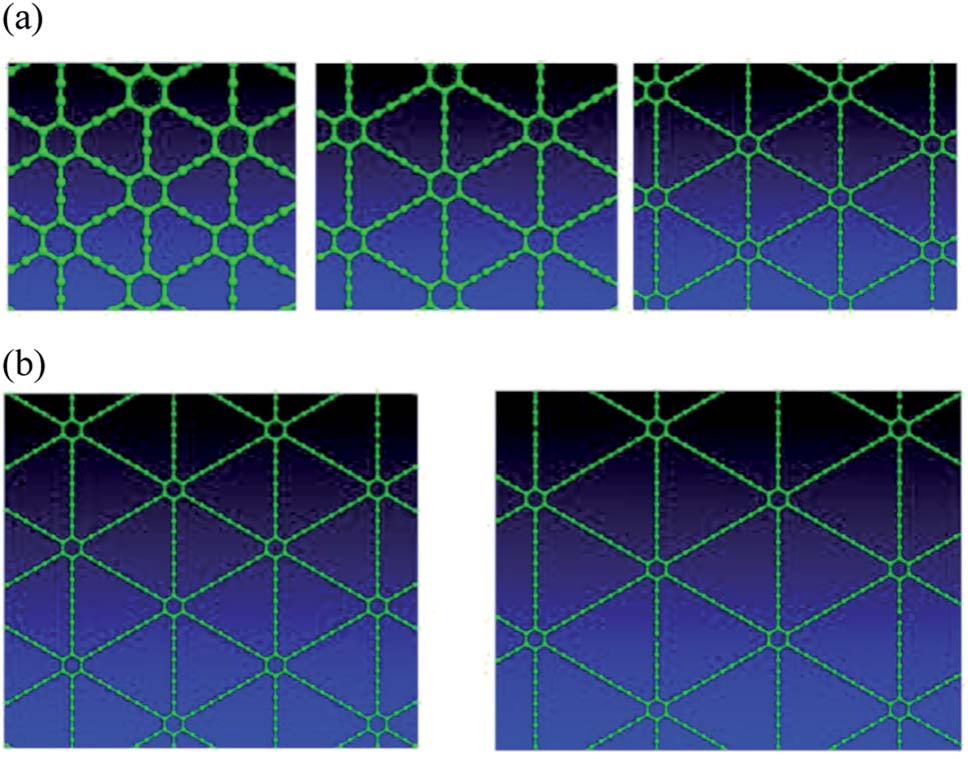
(a) Top views of γ-type graphyne-*n* varieties, where *n* indicates the number of carbon–carbon triple bonds in a link between two adjacent hexagons. Graphyne is graphyne-1 (left), graphdiyne is graphyne-2 (middle), and graphyne-3 (right); (b) top views of γ-type graphyne-4 (left) and graphyne-5 (right) 2D nanostructures.

Graphyne exists in a planar form, and is predicted to have high mobility carriers, and impressive thermal/phonon properties. In addition, the sp–sp^2^ multi-hybridized bonding character results in a number of possible graphyne allotropes, as shown in Fig. 1. Wet chemistry methods have recently been developed to build carbon nanomaterials, including sp–sp^2^ multi-hybridized graphyne systems, from the bottom up with atomic precision.^19,20^ Because of the large number of allotropes, graphyne provides a target-rich environment for synthetic organic efforts to have a positive impact on materials science. Our effort would be to find suitable ways to grow graphyne, create test structures, and determine electronic properties,^21^ proving or disproving the promise of this material, which has had almost no prior work^22^ done on it to realize useful electronic devices, with the exception of a mechanochemistry method for γ-graphyne.^23^

It is mentioned here that theoretical DFT calculations on both armchair and zigzag graphdiyne nanotubes have been done, providing estimates of bandgaps as a function of chirality and diameter, with possible higher conductivities of the carriers in zigzag nanotubes.^24^ Of course, the issue with planar sheets of single atomic constituent atoms not necessarily possessing useable bandgaps is less of an issue for nanotubes, which generally have bandgaps. Our past DFT simulations have examined some aspects of nanotubes and nanowires,^25,26^ which is why this paper focuses on single planar sheets for transistors. Of interest for better understanding this problem with finding useable bandgaps, and some of the problems with single atomic sheet materials, is the suggestion in a paper that borophene and graphyne be considered as potential materials to confront the zero-bandgap problem of graphene and other single atomic sheet materials plagued by zero bandgaps.^27^ Additionally, a very interesting study was recently conducted for graphyne under tension and compression deformation, providing electronic band structures, density of states (DOS), differential charge density plots, bond lengths and bandgap values.^28^ It is very undesirable to process transistors with intentional stresses, which can generate many defects, for initially fabricated electronic transistors using single atomic sheet materials, such as borophene and graphyne. However, specialized applications could be envisioned, as stress in certain devices and HEMT compound transistors have been employed to advantage.

In order to attain the sp–sp^2^ hybridization that is needed to grow graphyne, it was theoretically modeled^29^ that the growth conditions must be in the carbon-deprived regime instead of the C-rich regime which is typical for graphene growth. Therefore, the growth conditions must be modified from the standard growth of graphene. Growth technique variables that can be used to create sp–sp^2^ bonding include growth temperature, addition of precursors, and post growth processing. Graphyne could be grown using wet chemistry by coupling suitable small-molecule precursors *via* standard palladium-catalyzed cross-coupling reactions as well as *via* alkene and alkyne metathesis methods. Current homocoupling methods have already succeeded in bottom-up organic synthesis of graphene nanoribbons with precise edge structures, nitrogen and boron dopant placement, and the sp–sp^2^ graphdiyne allotrope.^17,18^ Cross-coupling will extend the synthesis of these materials by allowing the use of multiple precursor molecules to fabricate one structure. Additionally, alkene and alkyne metathesis,^29^ familiar to the synthetic organic community, have been severely underutilized in the synthesis of 2D carbon crystals such as graphyne.

Here we not only reexamine ordinary graphyne nanostructures which occur as one-atom-thick planar sheets of sp and sp^2^ bonded carbon atoms arranged in a crystal lattice with a lattice of benzene rings connected by acetylene bonds, but also variants with more than single acetylene bonds. Graphyne consists of a mixed hybridization sp^*n*^, where 1 < *n* < 2; graphene and graphite (considered pure sp^2^) and diamond (pure sp^3^). Here we extend the definition of graphyne to the notation graphyne-*n*, where *n* represents the number of acetylene bonds. Ordinary graphyne is in this system, graphyne-1. The synthesis of graphdiyne, which is graphyne-2, was reported as a 1 mm film on a copper surface.

Furthermore, there are other applications and physical possibilities of control, as it appears likely in either pristine α-, β-, or γ-graphyne or its substituted doped versions with one or two N or B atoms per unit cell,^30^ removing or adding electrons compared to C atoms, that one can tune from a bandgap point in *k*-space to a Dirac point, using an electric field applied through a gate voltage (which moves the Fermi level). This is a feature available in no other 2D material, and would provide new electronic possibilities. Also optical^31^ and magnetic^32^ control is available, heretofore, of significantly larger magnitude than in graphene. For example, the introduction of a single atom vacancy per unit cell in graphyne, can induce a magnetic moment of 1.1–1.3*μ*_B_ in α- and β-graphyne, and 1.8*μ*_B_ in γ-graphyne.

Finally, although not focused on here, there are numerous catalytic and energy conversion and storage possibilities with the graphyne family. We will summarize those here for completeness, in case the reader would wish to explore those related materials science, nanoscience and chemical possibilities. In the general area of catalysis, a discussion of strain bandgap effects, catalysis, use in batteries and more is available in ref. 33; for ammonia synthesis *via* graphdiyne and its use in photocatalysis, refer to ref. 34 and 35; and for atomically dispersed zerovalent molybdenum atoms on graphdiyne, see ref. 36. In the general area of energy storage and fuel cells, for lithium ion storage using chlorine substituted graphdiyne look at ref. 37, for ultrathin nanosheet graphdiyne grown on Cu nanowires and use as Li-ion battery anodes refer to ref. 38, and for aminated graphdiyne thin films for methanol fuel cells refer to ref. 39.

There is substantial literature on graphdiyne as a more advanced material, regarding its preparation and related areas. For information purposes, it is interesting and useful to provide the reader here with some references in this area which were not covered above. These treatments cover graphdiyne and its assembly structures, synthesis and properties, 2D carbon-graphdiyne, fundamentals and applications of graphdiyne, preparation of graphdiyne and its derivatives, synthesis of boron-graphdiyne and use in sodium storage, and modification of carbon atoms in graphdiyne using organic sulfur.^40–46^ There is some indication that preparation of graphdiyne may be more feasible or more readily available for chemical synthesis than basic graphyne.

## 2. The atomic structures

Graphyne allotropes of the γ-type will be examined in this work. These allotropes may be denoted by graphyne-*n*, where *n* indicates the number of carbon–carbon triple bonds in a link between two adjacent hexagonal benzene rings. Clearly, graphyne allotropes insert these triple bonds into graphene to create the new 2D material. Fig. 1(a) shows the atomic structures of graphyne-1, graphyne-2, and graphyne-3, where *n* has been chosen to be *n* = 1, *n* = 2, and *n* = 3. Fig. 1(b) shows the atomic structure of graphyne-4 (left) and graphyne-5 (right).

## 3. Structural relaxation electron density plot

**Fig. 2:**
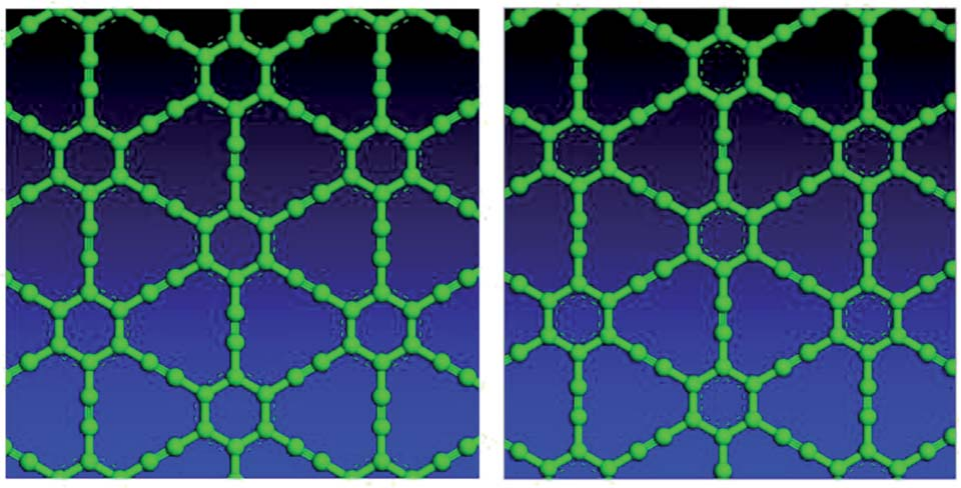
Top views of optimized γ-type graphyne-1 structures using LDA (left) and GGA (right).

Variable-cell structural optimization using the Broyden–Fletcher–Goldfarb–Shanno (BFGS) quasi-Newton algorithm was used to relax both the cell and the internal coordinates. The relaxed structures from both local density approximation (LDA) and generalized gradient approximation (GGA) calculations belong to the *P*6/*mmm* space group and are shown in Fig. 2. The volume of the LDA relaxed structure is 3.3% smaller than that of the GGA.

**Fig. 3:**
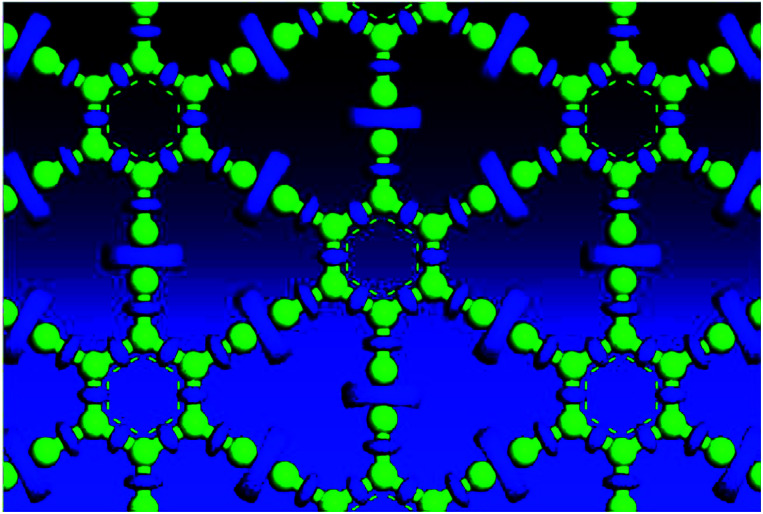
Electron localization function plot for a GGA optimized structure, top view.

Electron localization function (ELF) is a measure of the likelihood of finding an electron in the neighborhood space of a reference electron located at a given point and with the same spin. It is the electron charge density compared to the uniform electron gas case, and as such is a unitless number. This number is 0.00444743, and produces donut shaped objects, the size indicating the electron distribution. One sees in Fig. 3 much larger donuts between the triple bonded carbon atoms than the single bonded carbon atoms. Fig. 3 shows the plot of a GGA optimized structure, with the C–C bond length in the benzene ring being 1.444 Å; C–C partial double-bond length being 1.415 Å, and C–C triple bond length being 1.219 Å.

For the interested reader, ELF can be understood from its original Hartree–Fock derivation, using an energy density function *D*_*σ*_(**r**) with **r** being the spatial location for a spin *σ*.^47^ It is given by the formula

1 *D*_*σ*_(**r**) = *τ*_*σ*_(**r**) − [*∇ρ*_*σ*_(**r**)]^2^/[4*ρ*_*σ*_(**r**)]

Here *ρ* is the electron density for a particular spin *σ*, and the first term on the right hand side of the equation is the kinetic energy density *τ*, the second term a boson-like energy density, so their difference is the net fermionic contribution. Where electrons are highly localized, one expects *D*_*σ*_(**r**) to be small. Because such measurements as in (1) are not unique, it is compared to the uniform electron density which is *D*^0^_*σ*_(**r**),

2 *D*^0^_*σ*_(**r**) = 3(6π^2^)^2/3^[*ρ*_*σ*_(**r**)]^5/3^/5

Ratio of the energy densities in (1) and (2) yields a dimensionless quantity

3 *χ*_*σ*_(**r**) = *D*_*σ*_(**r**)/*D*^0^_*σ*_(**r**)

which is the relative energy density function. Electron localization as a function of spatial location **r** is then given by a mapping into the range 0 ≤ ELF(**r**) ≤ 1, using the expression

4 ELF(**r**) = [1 + {*χ*_*σ*_(**r**)}^2^]^−1^

Note that for perfect localization when *D*_*σ*_(**r**) = 0, relative density is *χ*_*σ*_(**r**) = 0 which is also null, making ELF(**r**) = 1, as desired and makes physical sense. For the uniform electron gas case when *D*_*σ*_(**r**) = *D*^0^_*σ*_(**r**), ELF(**r**) = 1/2. Extension of this approach to DFT has been done.^48^

## 4. Electronic band structure calculations

**Fig. 4:**
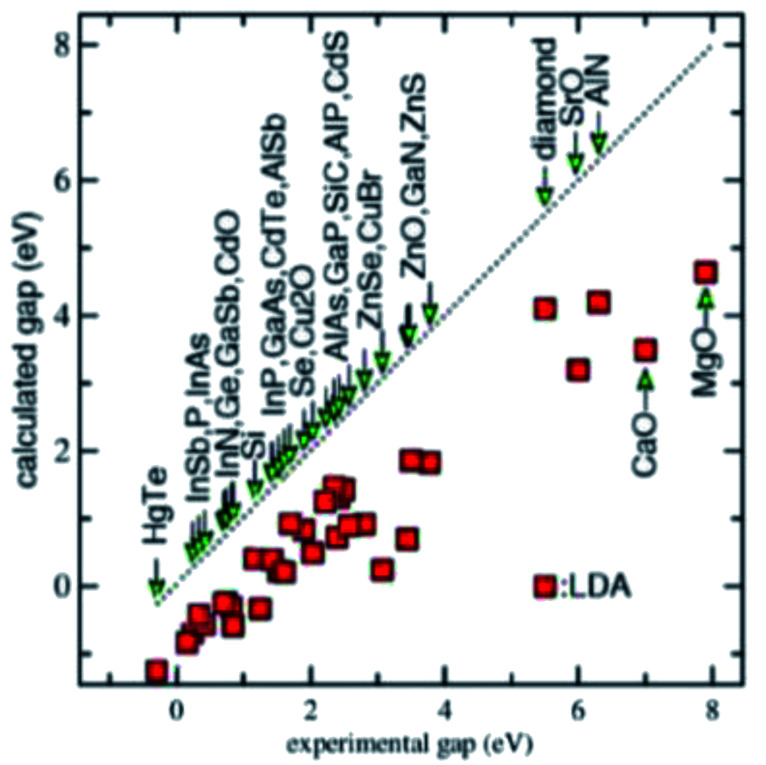
Infamous bandgap problem for LDA and GGA.

One key technical issue for the development of these crystalline 2D semiconductors is to be able to predict the accurate band structure and especially the band gap. The accuracy of the density functional theory (DFT) calculations strongly depends on the exchange-correlational functional, while the widely used local density approximation (LDA) and generalized gradient approximation (GGA) generally underestimate the band gap significantly.^49^ This is seen in Fig. 4. The various DFT exchange-correlational functionals have these known characteristics: the LDA functional depends only on the local density; GGA functional depends on local density and its gradient; meta-GGA functional depends on density, its gradient, and its second derivative; hybrid functional mixes in Hartree–Fock exchange. In Schilfgaarde *et al.*,^50^ the LDA behavior as well as the random phase approximation (RPA) or GW approximation of Hedin are shown.^51^ Here *G* stands for Green's function and *W* is the screened Coulomb interaction. Improvements came with Hybertsen and Louie^52^ employing the LDA eigenfunctions to generate the GW self-energy *Σ* = *iGW*. The approach in ref. 50, which is a modification of a full self-consistent GW method (full SCGW), which they refer to as the quasiparticle self-consistent GW (QSGW) method, results in accurate predictions of excited-state properties for a large number of weakly and moderately correlated materials. We won't belabor these points further, as long as one is aware of these intricacies of calculations.

All the current calculations are performed using HPC computer Mustang at AFRL DSRC. The quantum code employed on this supercomputer is quantum espresso, an integrated suite of open-source computer codes. Here we use it for electronic-structure calculations and materials modeling at the atomic level of detail. Quantum espresso can be used to examine quantum transport, structural optimization, molecular dynamics, electrochemistry, particular boundary conditions, response functions, potential energy surfaces, and spectroscopic behavior, all near the ground state of the atomic system. It is based on density-functional theory, a plane wave basis set, and pseudopotentials. For the GGA calculations, we use PBE potential, with a kinetic energy cutoff of 360 Ry for charge density and potential, *k* point mesh of 12 × 12 × 1, and convergence energy criteria set at 1.0 × 10^−4^ Ry.

**Fig. 5:**
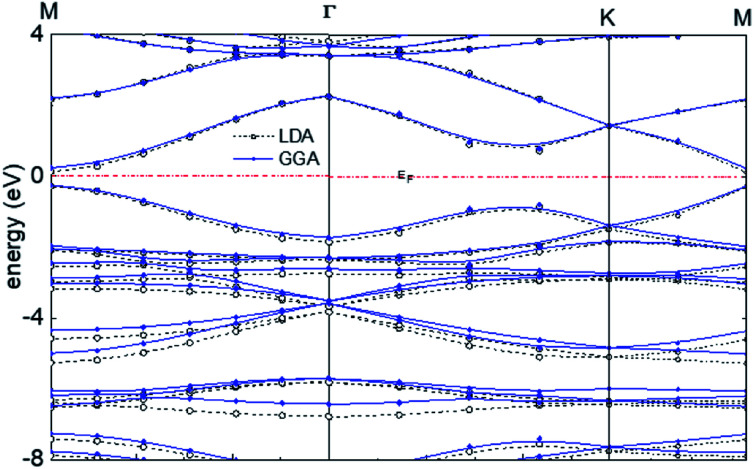
Calculated band structure of graphyne-1 using both LDA and GGA exchange-correlational functionals. The allotrope type is γ-graphyne.

The calculated band structure of graphyne-1 for the γ allotrope, that is, γ-graphyne, using both LDA and GGA exchange-correlational functionals is shown in Fig. 5, providing the energy *ε*(*k*) *vs. k* band structure through symmetry points in the Brillouin zone, *M*, *Γ* and *K*. LDA and GGA calculated band structures show reasonable agreements, especially close to the Fermi level. A direct band gap occurs at the *M* point in the Brillouin zone, with the gap of 0.49 eV for GGA and 0.411 eV for LDA.

**Fig. 6:**
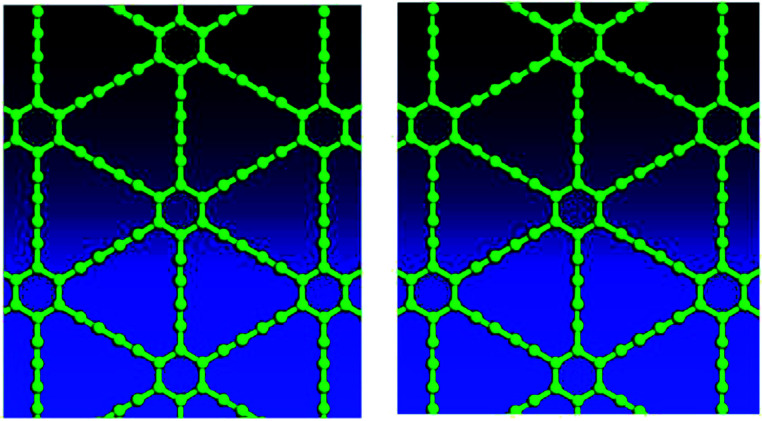
Top views of optimized γ-type graphyne-2 structures using LDA (left) and GGA (right). Variable-cell structural optimization using the Broyden–Fletcher–Goldfarb–Shanno (BFGS) quasi-Newton algorithm to relax both the cell and the internal coordinates.

**Fig. 7:**
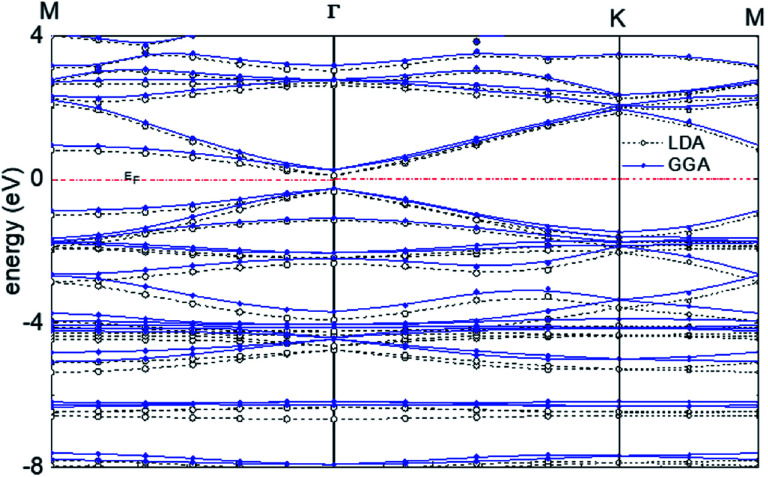
Calculated band structure of graphyne-2 using both LDA and GGA exchange-correlational functionals. The allotrope type is γ-graphyne.

For graphyne-2, again in the γ allotrope form, a variable-cell structural optimization using the Broyden–Fletcher–Goldfarb–Shanno (BFGS) quasi-Newton algorithm is used to relax both the cell and the internal coordinates. See Fig. 6. Both relaxed structures belong to the *P*6/*mmm* space group. The volume of the LDA relaxed structure is 2.9% smaller than that of the GGA. The calculated band structure of graphyne-2 using both LDA and GGA exchange-correlational functionals is shown in Fig. 7. LDA and GGA calculated band structures show reasonable agreements, especially close to the Fermi level. A direct band gap occurs at the *Γ* point in the Brillouin zone, with the gap of 0.515 eV for GGA and 0.437 eV for LDA.

To put everything in perspective from an absolute value of the bandgap, examine Table 1 reproduced from ref. 29 (superscripts a–e in the first column are explained in ref. 29). 40 different solid-state materials including C, Si, Ge, SiC, *etc.* are shown. LDA and GGA underestimate the band gap significantly; meta-GGA functional TPSS shows slight improvements; hybrid functional HSE improves the predicted band gap significantly. In this table the first two listings ME and MAE stand for, respectively, the mean error (ME) and mean absolute error (MAE).

**Table 1:** Bandgap error (eV) for 40 solid state materials

Solid	LSDA	PBE	TPSS	HSE
ME^a^	−1.14	−1.13	−0.98	−0.17
MAE^b^	1.14	1.13	0.98	0.26
rms^c^	1.24	1.25	1.12	0.34
Max (+)^d^	—	—	—	0.32
Max (+)^e^	−2.30	−2.88	−2.66	−0.72

## 5. Conclusions

In this work, two acetylene γ-allotropes of graphyne were studied and their band structures obtained. These two allotropes were γ-graphyne-1 and γ-graphyne-2, with respectively, one and two acetylene triple bonded carbon atom pairs connecting the benzene ring lattice. Two standard DFT techniques were used to assess whether or not these two allotropes present finite bandgaps which may be usable for electronics. What was found were nominal bandgaps of about 0.5 eV, roughly half of what silicon presents. Because the LDA and GGA techniques used for the high performance supercomputer calculations, are known to most likely underestimate bandgaps as discussed, we are fairly certain that useful bandgaps for electronic devices such as transistors will be found when experimentally preparing these materials. Thus we expect the bandgaps *E*_g_ to be 0.4 ≲ *E*_g_ ≲ 1.5 eV. Such bandgaps can be expected to be suitable for low and medium power electronics.

Future work is planned for investigating the effects of hybrid functional use in the first principles calculation of the electronic band structure for various graphyne-*n* orders. This should improve bandgap underestimation of *E*_g_ values obtained here, allowing an even more refined calculated quantity.

## Conflicts of interest

There are no conflicts to declare.
